# Letter regarding “Effect of synbiotic supplementation on immune parameters and gut microbiota in healthy adults: a double-blind randomized controlled trial”

**DOI:** 10.1080/19490976.2023.2262618

**Published:** 2023-09-27

**Authors:** Yongjian Lin

**Affiliations:** Department of Gastrointestinal and Gland Surgery, The First Affiliated Hospital of Guangxi Medical University, Nanning, China

Dear Editor:

We read with interest the study by Xiaoqin Li et al. that evaluated the effectiveness of probiotic supplementation on immune parameters and gut microbiota in healthy adults (a randomized controlled trial).^1^ This trial showed that synbiotics favored immune parameters and these immune parameters were associated with synbiotic-induced microbial changes, which altered the microbial enterotypes. However, the stratification criteria, statistical conclusions and sample comparisons might limit the generalizability of the findings. After carefully reading this article, we have some suggestions as follows.

First, as the authors described in the Study design, “Eligible participants were equally and randomly assigned into two groups using computer generated random numbers after stratification by gender and BMI (<24 or ≥24 kg/m^2^) without a predetermined block size”. There were no significant differences between the two groups at baseline, which is inconsistent with real-world individual polymorphism, and we suggest introducing propensity score matching (PSM) to balance baseline characteristics.^2^ Next, the World Health Organization (WHO) classified body mass index (BMI) into four categories: BMI < 18.5, 18.5–24.9, 25.0–29.9, and ≥30 kg/m^2.3^ Therefore, if the trial stratified participants’ BMI according to the WHO’s criterion, the information gained would be more comprehensive and the results reliable.

Second, the results of this trial suggest that synbiotic supplementation resulted in statistically significant reductions in C-reactive protein (CRP) and interferon-gamma (IFN-γ) levels at both week 4 and week 8. While describing the results as “these reductions were greater than those observed in the placebo group (*P* = .088 or .058 for CRP),” based on comparative results, the authors concluded that “synbiotic supplementation decreased pro-inflammatory biomarkers (CRP and IFN-γ)”. However, we found that the *P* value was not < .05 as the primary outcome with inconsistent result of IFN-γ. The above is not sufficient for conclusion, but the authors did not explore the reasons and discussion. Also, we observed that plasma interleukin (IL)-10 remained unchanged in the synbiotic group and decreased by 16% in the placebo group at week 8 (*P* < .001). The authors then determined that “there was a statistically significant increase in IL-10 levels in the synbiotic group compared to the placebo (*P* = .008)”. Since no significant effect of probiotics on IL-10 was observed in 22 previous clinical trials in healthy populations (heterogeneity of 75.7%),^4^ and systematic evaluations and meta-analyses suggested that IL-10 was unchanged in health, this result seems to be applicable only for short follow-up times and generalized linear mixed model assessments. We recommend that the authors follow up all patients until after 12 weeks and perform complete statistics to refine the results, which otherwise are not sufficient to support such a generalized conclusion.

A total of 106 patients were included in this article, which is a small sample study. This paper would have been improved if statistical efficiency or/and power could have been calculated. Furthermore, the interaction is an action in which two or more objects affect each other and play an essential role in this paper.^5^ It is generally recognized that co-morbidities and the administration of other medications may have an impact on clinical outcomes. However, although many of the characteristics are described in Table 1, subgroup analyses were not performed based on factors such as age, blood pressure and body composition. Therefore, this paper may be more rigorous if the interactions are put into practice.

**Table 1.** Consumption per capita and average beer price.

Variable	Control group (n = 115)	RS group (n = 115)	F or X^2^ (p value)
Gender(male/female)	62/53	66/49	0.3 (0.60)
Age (years)	26.2 ± 6.6	26.1 ± 5.9	0.01 (0.92)
Education (years)	11.9 ± 2.3	12.4 ± 2.5	2.2 (0.14)
Age of onset (years)	24.0 ± 5.8	23.6 ± 5.2	0.2 (0.67)
Duration of illness (years)	2.2 ± 1.6	2.5 ± 1.8	1.8 (0.19)
Prolactin (ug/ml)	254.7 ± 54.9	259.1 ± 59.5	0.6 (0.44)
P	24.9 ± 6.2	23.9 ± 6.3	1.4 (0.24)
N	28.9 ± 6.1	30.2 ± 7.2	2.3 (0.14)
G	58.3 ± 8.8	59.9 ± 8.8	1.7 (0.19)
PANSS total score	112.2 ± 12.5	114.0 ± 13.4	1.2 (0.27)
HAMD total score	24.3 ± 4.8	24.8 ± 5.7	0.4 (0.51)
PSP total score	34.9 ± 5.2	34.2 ± 3.6	1.7 (0.20)
CGI-S total score	5.9 ± 0.7	5.8 ± 0.6	0.1 (0.77)

In conclusion, we thank the authors for this excellent work, which provides direct evidence for personalized supplementation of immune regulation in healthy individuals. However, we believe that the conclusions of the study would have been stronger if the previously mentioned issues had been further addressed.
